# Identification of immune microenvironment subtypes that predicted the prognosis of patients with ovarian cancer

**DOI:** 10.1111/jcmm.16374

**Published:** 2021-03-06

**Authors:** Xinjing Wang, Xiaoduan Li, Xipeng Wang

**Affiliations:** ^1^ Department of Gynecology and Obstetrics XinHua Hospital Shanghai JiaoTong University School of Medicine Shanghai China; ^2^ Department of Gynecology Shanghai First Maternity and Infant Hospital Tongji University School of Medicine Shanghai China

**Keywords:** immunotherapy, ovarian cancer, prognosis, tumour microenvironment

## Abstract

Ovarian cancer (OC) is associated with high mortality rate. However, the correlation between immune microenvironment and prognosis of OC remains unclear. This study aimed to explore prognostic significance of OC tumour microenvironment. The OC data set was selected from the cancer genome atlas (TCGA), and 307 samples were collected. Hierarchical clustering was performed according to the expression of 756 genes. The immune and matrix scores of all immune subtypes were determined, and Kruskal‐Wallis test was used to analyse the differences in the immune and matrix scores between OC samples with different immune subtypes. The model for predicting prognosis was constructed based on the expression of immune‐related genes. TIDE platform was applied to predict the effect of immunotherapy on patients with OC of different immune subtypes. The 307 OC samples were classified into three immune subtypes A‐C. Patients in subtype B had poorer prognosis and lower survival rate. The infiltration of helper T cells and macrophages in microenvironment indicated significant differences between immune subtypes. Enrichment analyses of immune cell molecular pathways showed that JAK–STAT3 pathway changed significantly in subtype B. Furthermore, predictive response to immunotherapy in subtype B was significantly higher than that in subtype A and C. Immune subtyping can be used as an independent predictor of the prognosis of OC patients, which may be related to the infiltration patterns of immune cells in tumour microenvironment. In addition, patients in immune subtype B have superior response to immunotherapy, suggesting that patients in subtype B are suitable for immunotherapy.

## 1 INTRODUCTION

Ovarian cancer (OC) is the malignancy of the female reproductive system with the highest mortality rate.^1^, ^2^ The diagnosis of OC at early stage is difficult, and widespread pelvic and abdominal dissemination often occur at the time of diagnosis. Patients with advanced OC generally have poor prognosis due to resistance to conventional treatment. The 5‐year survival rate of patients with OC diagnosed as FIGO stage III and IV is only 20%.^3^

Tumour immune microenvironment can affect tumour metastasis.^4^, ^5^ OC microenvironment exhibits complex immune cell infiltration characteristics involving T cells, DC cells, macrophages and NK cells. Immune cells release immunoregulatory factors and interact with each other to influence the progression, prognosis and treatment responsiveness of patients with OC.^6^, ^7^, ^8^ Studies have reported that OC cells can regulate the differentiation of macrophages into M2 tumour‐associated macrophages in the microenvironment.^9^ Tumour‐associated macrophages can further mediate an imbalance in the T‐cell differentiation ratio in the microenvironment.^10^ M2 tumour‐associated macrophages can promote peritoneal metastasis of OC by accelerating angiogenesis.^11^

However, the correlation between immune microenvironment and prognosis of OC remains unclear. Therefore, this study aimed to explore prognostic significance of OC tumour microenvironment. We divided OC into three immune subtypes on the basis of clinical features according to the expression of immune‐related genes. We analysed immune subtypes of 307 samples from the cancer genome atlas (TCGA) database and 93 samples from the international cancer genome consortium (ICGC) database, OC patients with high immune and matrix scores in subtype B exhibited poor prognosis. Additionally, the infiltration of M1‐type macrophages and helper T cells decreased, whereas the infiltration of Treg‐type macrophages and M2‐type macrophages increased. The enrichment fraction of the IL‐6‐JAK‐STAT3 molecular pathway increased in the immune subtype samples of subtype B. However, the enrichment fraction of the KRAS‐down‐regulation pathway increased in A and C immune subtype samples with superior prognosis.

## 2 MATERIALS AND METHOD

### 2.1 Data acquisition

OC data set was selected from the TCGA, and the samples lacking survival data were excluded. Finally, the expression of 20 188 genes in 307 ovarian serous adenocarcinoma samples was analysed. Clinical information included stage, grade, lymph node metastasis and molecular subtype of samples (https://tcga.Xenahubs.net/download/tcga.ov.samplemap/hisekov2.gz). The patients' information was provided in Table S1.

The ICGC data set selected in the present study was transformed according to the GeneSymbol. The expression of the same GeneSymbol was combined, whereas the expression of 43 638 genes was obtained from 93 OC samples (http://DCC.icgc.org/releases/current/Projects/OV‐AU).

### 2.2 Hierarchical clustering

The distance between samples was calculated using hierarchical clustering. The nearest points were merged into the same class each time. Then, the distance between classes was calculated, and the nearest classes were merged into one large class until a single class was synthesized. The distance between classes was calculated using methods such as single linkage, complete linkage, average linkage and unweighted pair group method with arithmetic mean (UPGMA). The complete linkage was chosen as the calculation method, the calculated distance was Chebyshev distance, and the clustering algorithm constructed in the pheatmap package in R was used.

### 2.3 Survival analyses

Survival analyses were performed using survival and survminer packages in R and further visualized using the Kaplan‐Meier curve.

### 2.4 Immune score and matrix score

The immune and matrix scores of each OC sample were calculated by ESTIMATE (R package: estimate).

### 2.5 Evaluation of immune cell infiltration

CIBERSORT was used to normalize gene expression data to infer the infiltration ratio of 22 types of immune cells. To evaluate the reliability of deconvolution, CIBERSORT was used to calculate the P value for each sample, and the correlation analysis was performed after screening based on a *P* value of < 0.05.

### 2.6 Gene set variation analysis

Gene set variation analysis (GSVA) is a non‐parametric and unsupervised algorithm used to calculate the enrichment scores of specific gene sets in each sample without grouping samples in advance. GSVA transforms gene expression from an expression matrix characterized by a single gene to an expression matrix characterized by a specific gene set. GSVA was used to calculate the enrichment scores of different gene sets in each sample of TCGA, and correlation analyses were performed.

## 3 RESULTS

### 3.1 OC immunotyping

**FIGURE 1:**
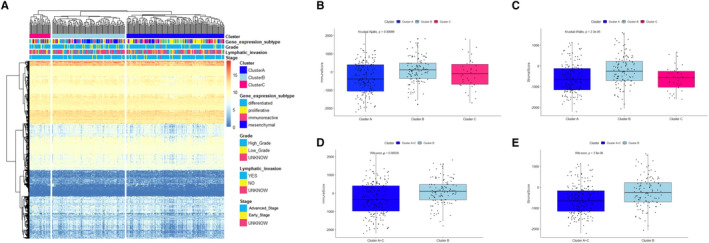
Immune clusters of OC were associated with immune score. (A) Cluster heat map of expression levels of 756 immune‐related genes in 307 samples. The right panel demonstrated the cluster grouping, four molecular subtypes, grade classification (G1 and G2 were low grade and G3 and G4 were high grade), lymph infiltration and stage classification (stage III and IV were advanced stage, and stage I and II were early stage). (B) Box plots of immune score of three subtypes of immune subtypes. (C) Box plots of matrix score of three subtypes of immune subtypes. (D) Box plots of immune score of subtype A + C and subtype B. (E) Box plots of matrix score of subtype A + C and subtype B

A total of 756 genes were filtered from 770 immune‐related genes in the TCGA database. Based on the expressions of 756 genes, hierarchical clustering analyses were performed on 307 genes in the TCGA database (complete, longest distance method, and Maximum and Chebyshev distance). Three immune subtypes of OC (clusters A, B and C) were obtained, and the expression heat map (Figure 1A) was constructed according to clinical phenotype.

The estimate in R package was used to calculate the immune and matrix scores of each sample in the TCGA data set, and the immune score (Figure 1B) and matrix score (Figure 1C) were compared between the three subtypes of OC samples with different immune subtypes. Comparison of immune scores (Figure S1A‐C) and matrix scores (Figure S1D‐F) in pairwise groups indicated that the immune and matrix scores of OC samples with immune subtypes in subtype B were significantly higher than those in subtype A and C; however, no significant difference was observed in the scores between subtype A and C. Therefore, subtype A + C was compared with subtype B (Figure 1D,E), and subtype B (n = 117) had significantly higher immune and matrix scores than subtype A + C (n = 191).

### 3.2 Immunotyping was related to the prognosis of patients with OC

**FIGURE 2:**
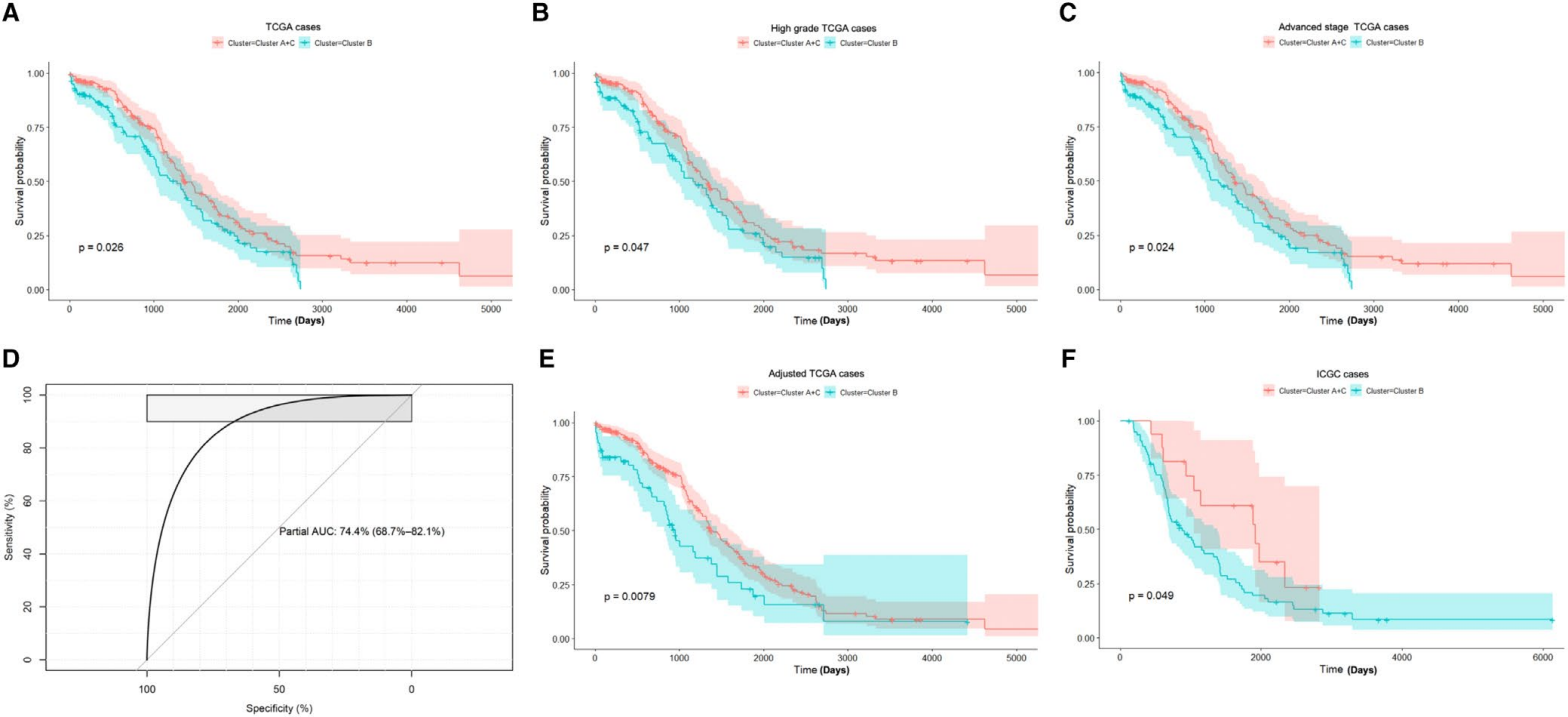
Immune clusters were associated with prognosis. (A) Kaplan‐Meier analysis of the difference in prognosis between cluster A + C and Cluster B, with survival time in days. (B) In 267 highly graded samples, the difference of prognosis between cluster A + C and cluster B was compared. (C) In 282 samples at advanced stage, the difference of prognosis between clusters A + C and cluster B was compared. (D) The binomial logistic regression classifier was constructed by using the R package‐glmnet; ROC curve was drawn based on the samples of cluster B. The overall AUC of the model was 0.743, and the prediction accuracy of Cluster B samples was 80.95%. (E) The survival analysis and verification of the prediction model based on TCGA data. (F) The survival analysis and verification of the prediction model based on external test set data (ICGC data)

Kaplan‐Meier analysis was used to determine the correlation of immunophenotyping with survival and prognosis of patients with OC. The overall survival of patients in subtype B was significantly shorter than that of patients in subtype A and C (Figure 2A). Analyses of survival and prognosis of OC samples with high‐grade pathological type (G3, G4, n = 267) and advanced clinical stage (FIGO stage III and IV, n = 282) indicated that overall survival of patients in subtype B was significantly shorter than that of patients in subtype A + C (Figure 2B,C).

Based on the expression of 756 immune‐related genes, 90% of the sample data were randomly selected from TCGA to construct a model, and the remaining 10% data were used to verify the prediction results. The overall area under the curve of the model was 0.743, and the prediction accuracy of cluster B samples was 80.95% (Figure 2D). Based on the prediction model, the survival analyses and verification were performed on the TCGA and external data set (ICGC data) (Figure 2E,F). The number of samples in subtype B was reduced (from 116 to 63) by the model prediction. The survival log rank test indicated that this classification improved the significance of correlation between immune subtype grouping and survival prognosis, and the ICGC data analysis confirmed that overall survival of patients in subtype B was significantly lower than that of patients in subtype A + C.

**FIGURE 3:**
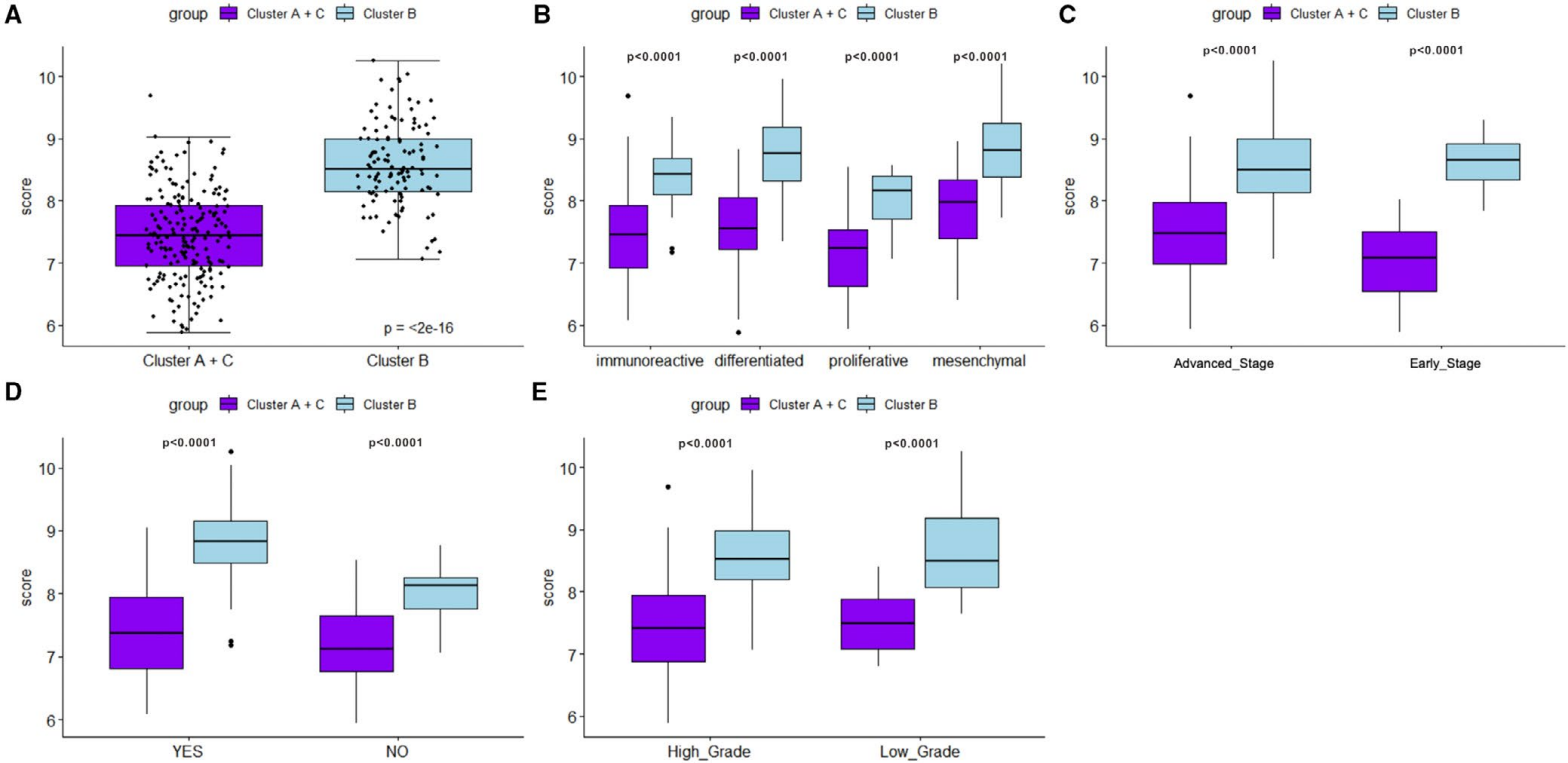
Immune clusters and clinicopathological features. (A) The prognostic risk of samples was calculated by using R package‐glmnet, and the overall prognosis was compared among samples in each immune subtype. (B) The prognosis risk of different immune subtypes was compared among four molecular subtypes. (C) The prognosis risk of different immune subtypes was compared among patients with varying clinical stages. (D) The prognosis risk of different immune subtypes was compared among patients with lymph node invasion or non‐invasion. (E) The prognosis risk of different immune subtypes was compared among patients with differing pathological grades

R package‐glmnet was adopted to calculate the prognostic risk of each immune subtype (Figure 3A). The prognostic risk of different immune subtypes was compared based on molecular types (Figure 3B), clinical stages (Figure 3C), lymph node invasion (Figure 3D) and pathological grades (Figure 3F). The results indicated that the prognosis of B immune subtypes in patients with various clinical and molecular phenotypes was significantly poor.

### 3.3 Immunotyping and immune cell infiltration

**FIGURE 4:**
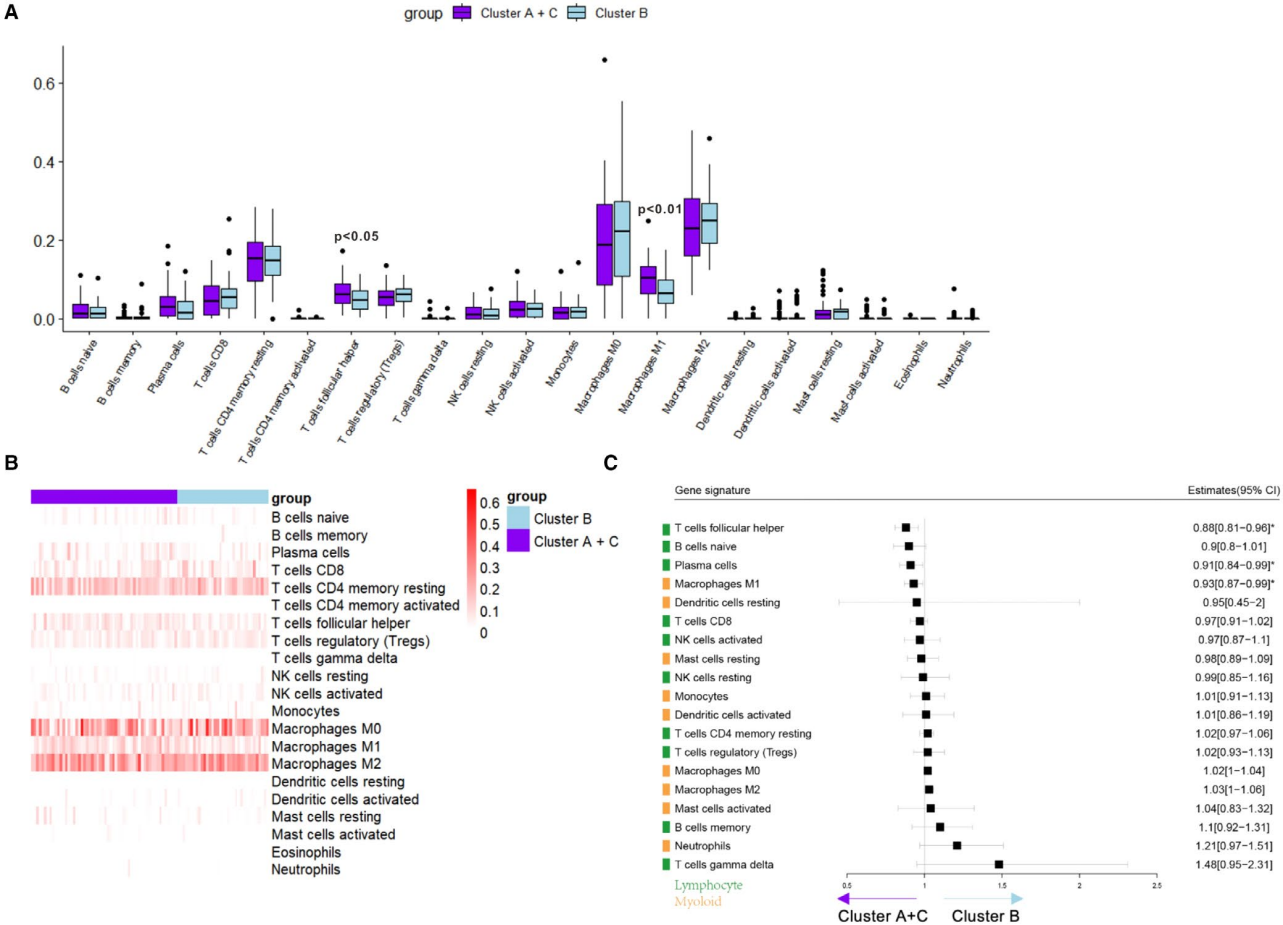
Immune clusters and immune cells infiltration. (A) CIBERSORT was utilized to estimate the infiltration ratio of immune cell types, and the infiltration of immune cells was compared in different immune subtypes. (B) The heat map was plotted according to the infiltration fraction of immune cells in each subtype. (C) The generalized linear model was established and a forest map was plotted according to the infiltration fraction of immune cells in each subtype

CIBERSORT was adopted to compare the correlation between lymphoid and myeloid infiltration. The infiltration ratio of 22 immune cells was calculated, and the differences in different immune subtypes were compared (Figure 4A). Then, a cluster diagram was plotted according to the infiltration value of each group of immune cells in different immune subtype samples (Figure 4B). By establishing a generalized linear model, a forest map was plotted to compare immune cell infiltration patterns in immune subtypes of subtype B (Figure 4C).

**FIGURE 5:**
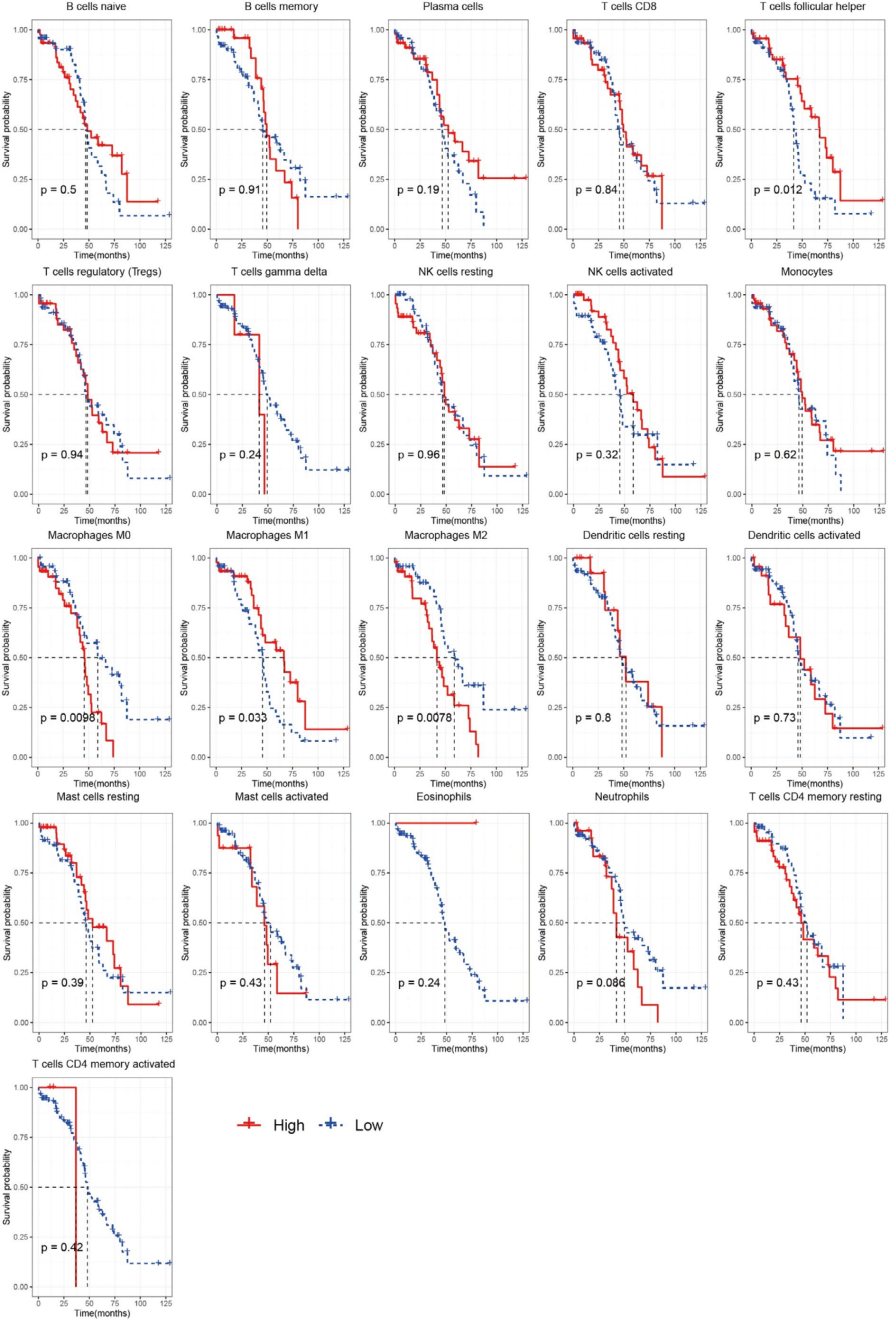
Immune cells infiltration was associated with prognosis. According to infiltration value of different immune cells, they were divided into two groups (high and low group), and the correlation between the infiltration of different immune cells and the survival and prognosis of patients was analysed

In immune subtypes of subtype B with poor prognosis, the infiltration of T cells follicular helpers and M1 macrophages decreased significantly, whereas the infiltration of M2 macrophages, regulatory T cells (Treg) and mast cells resting displayed an increasing trend. Furthermore, the correlation between the infiltration of immune cells and the prognosis of patients was analysed (Figure 5A). Survival analyses indicated that increased infiltration of M2‐type macrophages and decreased infiltration of M1‐type macrophages and T cells were associated with poor prognosis. Collectively, tumour microenvironment in subtype B immune subtype may promote tumour progression, and the infiltration pattern of immune cells in this immune subtype may be related to poor prognosis.

### 3.4 Analyses of immunosubtype‐related molecular pathways and immunotherapy reactivity

**FIGURE 6:**
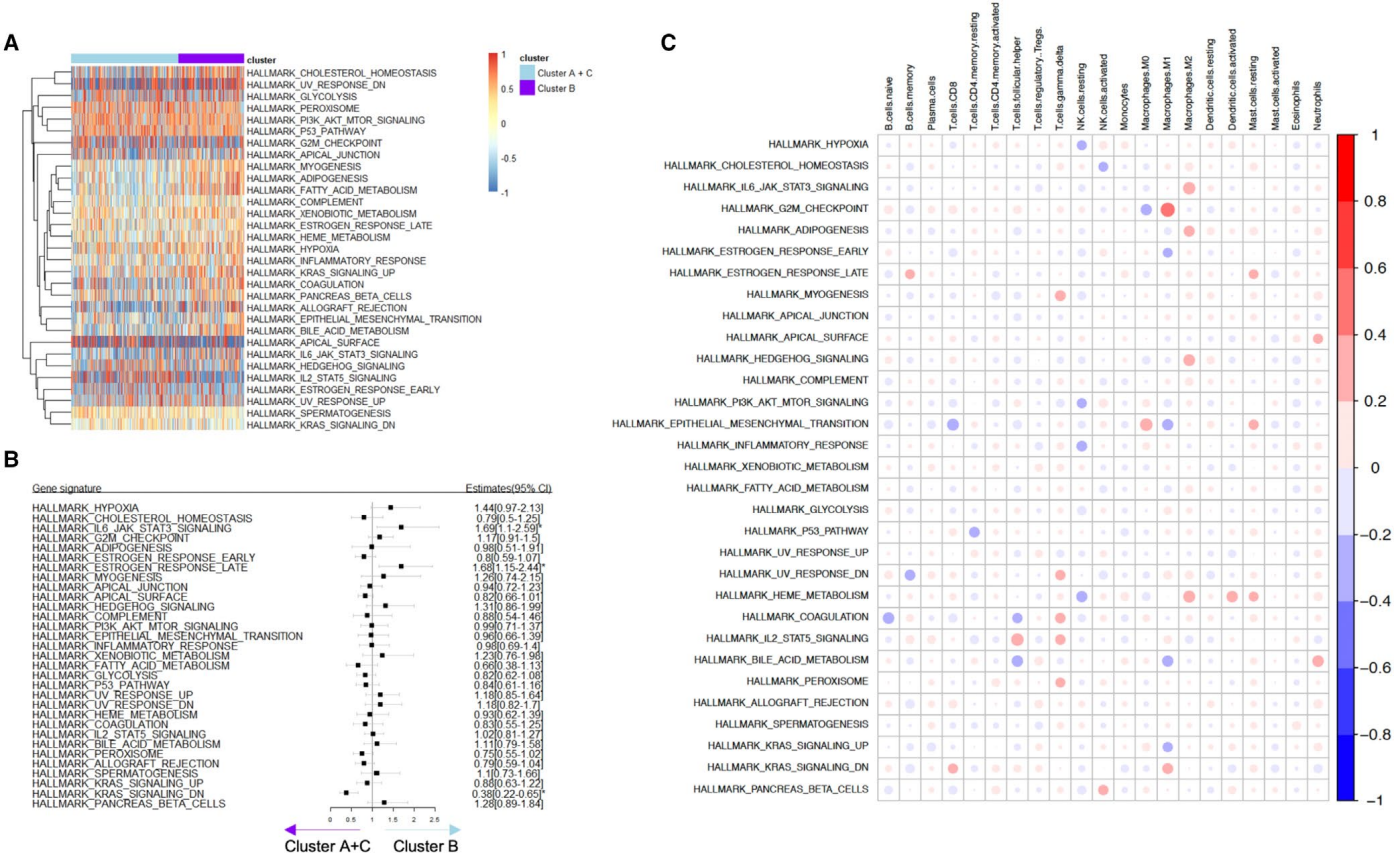
Immune clusters and phenotypes in ovarian cancer. (A) Using GSVA, the average gene set enrichment score was calculated in hallmark, and the score heat map was drawn based on different immune subtypes. (B) A generalized linear model was established, and a forest map was drawn. (C) Based on the infiltration of immune cells, the correlation between the enrichment fraction of gene set and the infiltration of immune cells was analysed

To further analyse the phenotype related to poor prognosis of patients with immune subtype B, GSVA was used to calculate the average gene set enrichment score of the gene set in hallmark, and a cluster map was constructed according to the grouping (Figure 6A). The scores of gene sets among subtypes were compared using the generalized linear model, and the forest map was plotted to analyse molecular pathway characteristics of immune subtypes in subtype B (Figure 6B). Pathways such as JAK‐STAT3 and oestrogen_response_late were significantly enriched in immune subtype B, whereas KRAS signalling pathway was remarkably enriched in immune subtype A + C.

Because immune subtypes are related to the infiltrating immune cell type and gene set signatures, we further determined the correlation between immune cell infiltration and gene set scores (Figure 6C). The results showed that the enrichment fraction of the JAK‐STAT3 pathway was positively correlated with M2 macrophage infiltration, and the enrichment fraction of the oestrogen response late pathway was positively correlated with the infiltration of resting mast cells. The enrichment fraction of KRAS signalling pathway was positively correlated with the infiltration of immune cells such as M1 macrophages and follicular helper T cells. Taken together, tumour microenvironment molecular pathway is consistent with immune molecular phenotype and immune cell infiltration pattern.

**FIGURE 7:**
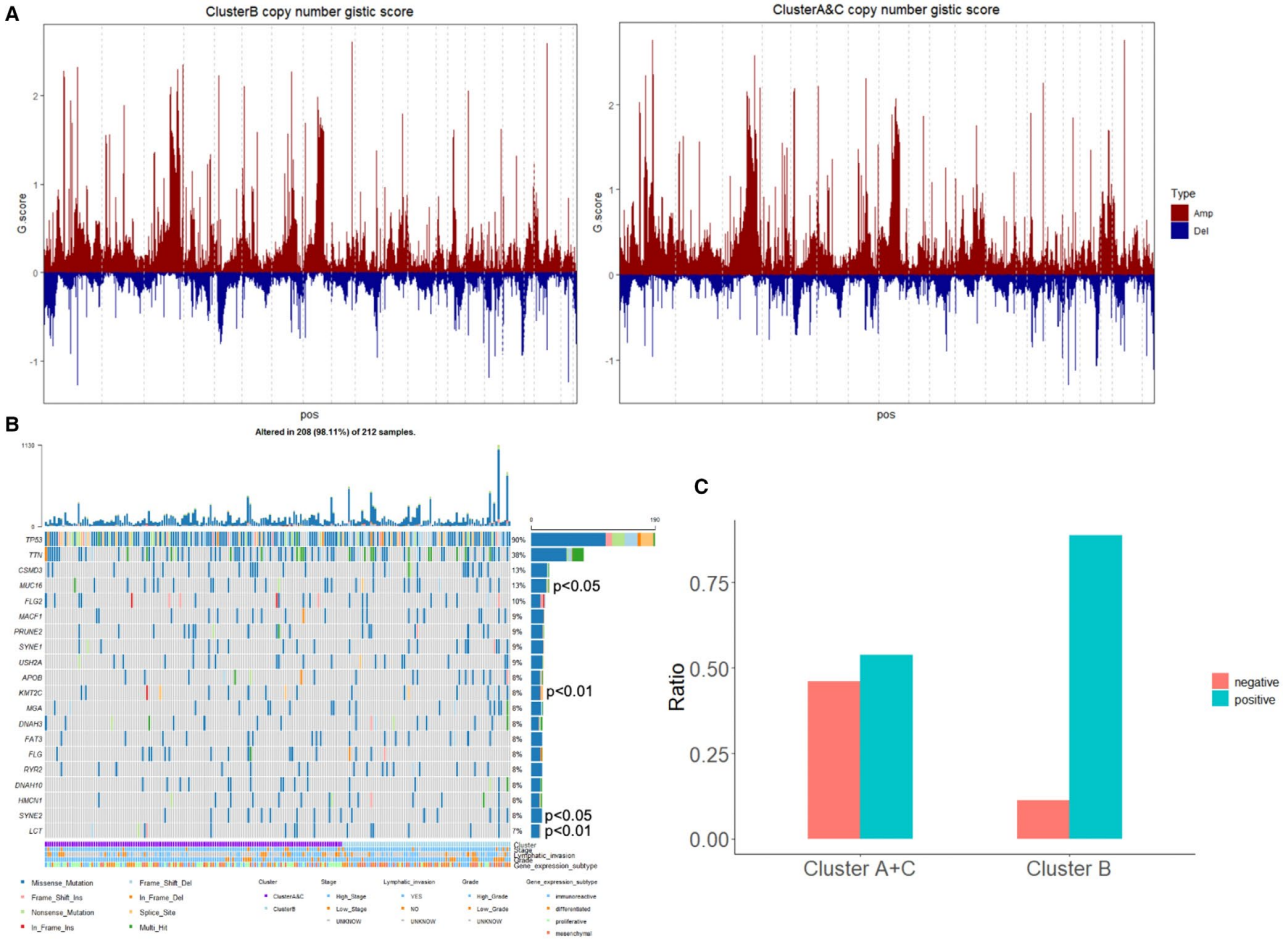
Gene mutations and immunotherapy effect of immune clusters. (A) According to the grouping of immune subtypes, CNV mutations of two groups of samples were analysed using GenePattern GISTIC_2.0. (B) The mutation information of OV data set was downloaded from GDC to screen 307 samples, the top 20 genes in overall mutation rate were selected, and the mutation information of samples was plotted by using R package‐maftools. (C) Using TIDE, the immunotherapy effect of patients with various immune subtypes was predicted

Furthermore, gene mutation patterns of different immune subtypes of 307 TCGA samples were analysed. The copy number variation (CNV) pattern of each group was obtained using the GenePattern GISTIC_2.0 (Figure 7A). R package‐maftools was utilized to infer the mutation information of samples (the first 20 genes selected, Figure 7B), and we found the differences in mutations of MUC16, KMT2C, SYNE2 and LCT between subtype B and subtype A + C. The missense mutation was the main type of mutation (Figure S2A,B). P53 exhibited the highest mutation rate of 88% (Figure S2C).

The immunotherapy effect was predicted using TIDE (http://tide.dfci.harvard.edu/) (Figure 7C), and the predicted immunotherapy effect in patients with immune subtypes in subtype B was superior.

## 4 DISCUSSION

Immune characteristics of tumour microenvironment is crucial in OC progression. Analyses based on public data sets like the Gene Expression Omnibus (GEO), The Cancer Genome Atlas (TCGA), InSilicoDB, curatedOvarianData, ArrayExpress and PubMed have shown that the immune cell infiltration signature and the immune‐related genes expression play important roles in the shape of immune microenvironment in ovarian cancer and breast cancer.^12^, ^13^ In the present study, we focused on immune subtype of OC according to the expression of immune‐related genes. OC data set was selected from the TCGA since the data set has relatively complete clinical information and larger patient sample size. And then a prediction model was established to determine whether a patient would have immune subtype with poor prognosis.

This study revealed a novel subtype of OC immune characteristics and showed that immune subtype B was related to poor prognosis in patients. The immune and matrix scores of tumour samples in subtype B increased significantly, and overall survival of this group decreased significantly regardless of the clinical stage, pathological grade, lymph node invasion and molecular classification of patients.

Furthermore, the accuracy of this model was verified using the external data set (ICGC). Moreover, we identified immune cell infiltration pattern and molecular pathway enrichment characteristics in tumour microenvironment of subtype B immune subtype samples.

Tumour‐associated macrophages (TAM) are crucial in OC progression, and the ratio of M1/M2 macrophages can be used as a prognostic indicator for OC patients.^14^ M1 macrophages can stimulate helper T cells to secrete cytokines such as IL‐12, IL‐23 and TNF‐α, which are related to the improvement in long‐term survival of OC patients.^15^ Some studies have suggested that helper T cells have significant anti‐tumour immunity, and the activation of helper T cells may be a novel method for improving tumour immunotherapy.^16^

In this study, we found that the infiltration ratio of M2 macrophages and Treg cells in B immune subtype samples exhibited an increasing trend. However, the difference was not significant, which may be due to the limited sample size. The tumour‐promoting function of M2 macrophages has been widely reported.^17^ Monocytes or macrophages in OC microenvironment can polarize to M2 macrophages, and M2 macrophages can further shape immune microenvironment that enhances OC progression.^10^, ^18^ Therefore, OC immunotherapy focuses on facilitating the transformation of M2‐type macrophages to M1‐type macrophages.^19^ Treg cells are considered to be immunosuppressive, and increased infiltration of Treg cells has been found to be generally associated with poor prognosis of patients with OC.^20^, ^21^

The enrichment analysis of immune subtype‐related molecular pathway indicated an increase in the enrichment fraction of IL‐6‐JAK‐STAT3 molecular pathway in B subtype, which was highly correlated with M2 macrophage infiltration. IL‐6‐JAK‐STAT pathway plays a significant role in the regulation of tumour immune microenvironment,^22^ and its activation is related to drug resistance of OC.^23^ A recent single‐cell sequencing study on high‐grade OC indicated that JAK–STAT pathway was abnormally activated in OC cells and small molecule inhibitors of this pathway are promising candidates for clinical application.^24^ Pathway analyses indicated that the enrichment scores of KRAS pathway increased in the immune subtype samples of subtype A and C with better prognosis. KRAS gene mutation and the activation of downstream pathway are significant molecular events in OC progression,^25^ which are significantly related to poor prognosis in patients with OC.^26^

Finally, we analysed the correlation between immune subtypes and immunotherapy. TIDE was used to predict the response of OC samples with different immune subtypes to immunotherapy. The results indicated that patients with immune subtype B with poor prognosis had superior response to immunotherapy. Since immunotherapy has not been widely developed in ovarian cancer, the patients' response to immunotherapy was predicted by TIDE analysis, which is a limitation of our research. It needs further study to confirm the correlation between immune subtypes and the response to immunotherapy in ovarian cancer patients received immunotherapy.

In conclusion, our study revealed that tumour immune subtypes can be used as an independent predictor for OC prognosis. Special tumour immune subtype might guide clinical treatment decision and improve the immunotherapy responsiveness of OC patients, thus providing a novel direction for the development of effective immunotherapy strategies.

## CONFLICT OF INTEREST

The authors confirm that there are no conflicts of interest.

## AUTHOR CONTRIBUTIONS

**Xinjing Wang:** Data curation (equal); Formal analysis (equal); Investigation (equal); Resources (equal); Software (equal); Writing‐original draft (equal). **Xiaoduan Li:** Data curation (equal); Formal analysis (equal); Investigation (equal); Validation (equal). **Xipeng Wang:** Funding acquisition (equal); Supervision (lead); Writing‐review & editing (lead).

## Supporting information

Fig S1‐S2

Table S1
