# Supplementary figures and images for: Identification of immune microenvironment subtypes that predicted the prognosis of patients with ovarian cancer
Source: J Cell Mol Med. 2021 Mar 6;25(8):4053–61. doi: 10.1111/jcmm.16374 (PMC8051724; doi:10.1111/jcmm.16374)

Figure S1

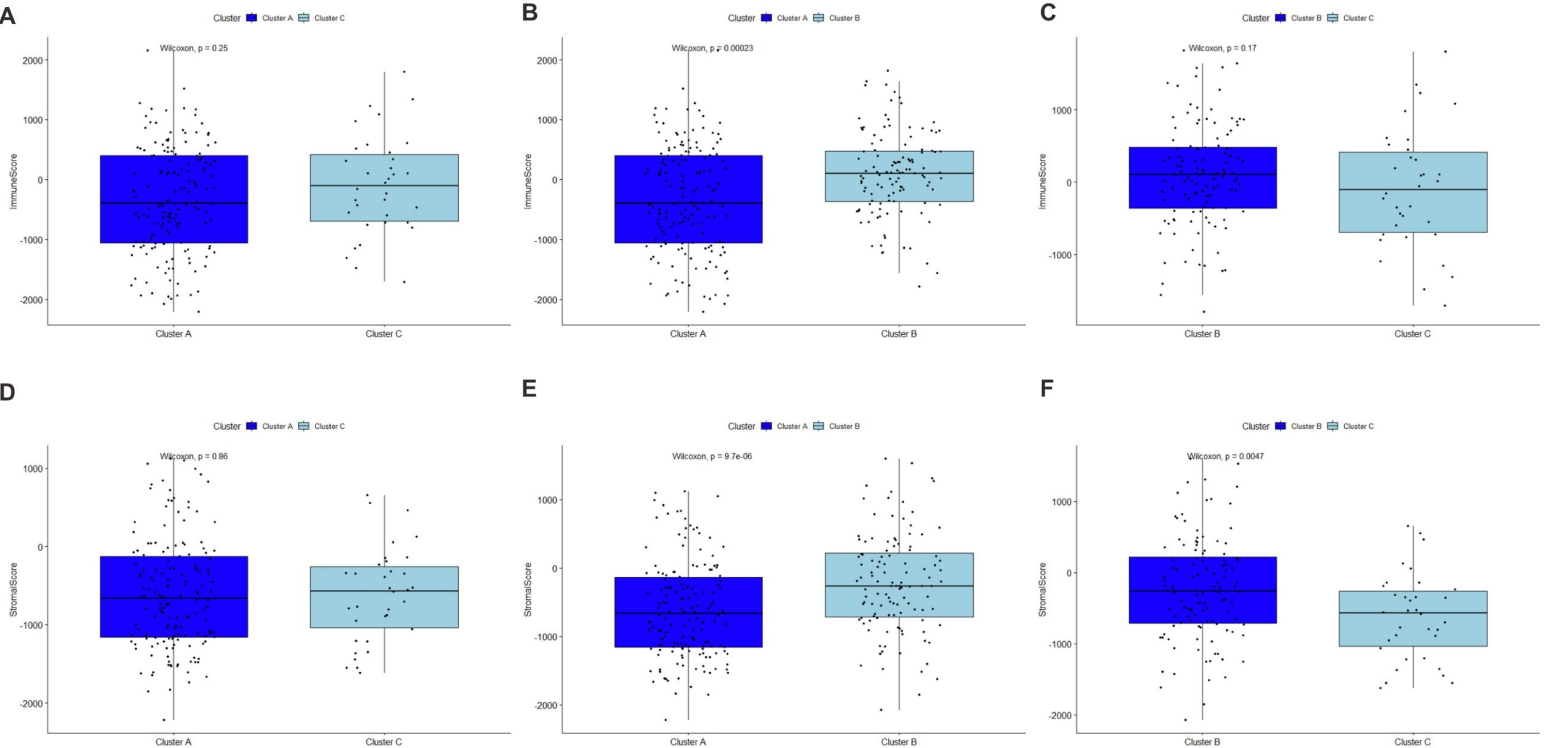

Figure S2

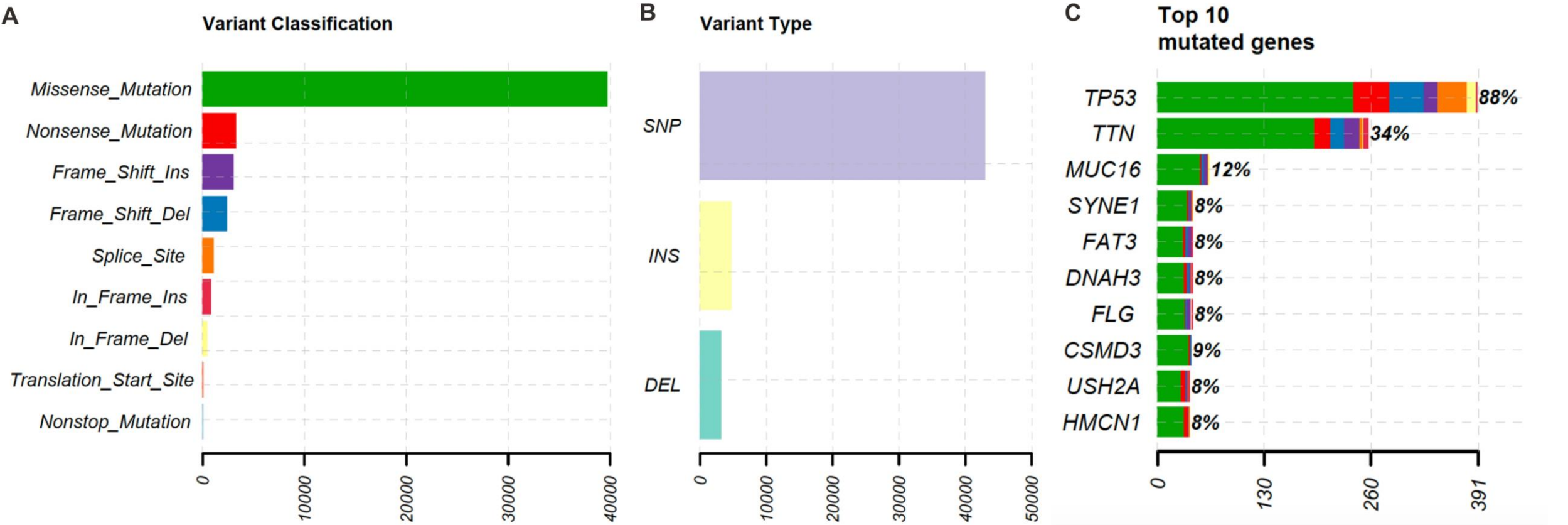

Supplement: Supplementary file 1 — Fig S1‐S2 [file JCMM-25-4053-s002.pdf]
