# Supplementary material for: Identification of immune microenvironment subtypes that predicted the prognosis of patients with ovarian cancer
Source: J Cell Mol Med. 2021 Mar 6;25(8):4053–61. doi: 10.1111/jcmm.16374 (PMC8051724; doi:10.1111/jcmm.16374)
Supplement: Supplementary file 2 — Table S1 [file JCMM-25-4053-s001.docx]

| Supplementary Table1. Clinical information of the OC patients in TCGA dataset | | | |
| --- | --- | --- | --- |
| Sample ID | age | stage | grade |
| TCGA-61-1910-01 | 56 | Stage IIC | G3 |
| TCGA-61-1728-01 | 59 | Stage IV | G3 |
| TCGA-09-1666-01 | 57 | Stage IIIC | G3 |
| TCGA-24-1469-01 | 71 | Stage IIIC | G3 |
| TCGA-04-1348-01 | 44 | Stage IIIB | G3 |
| TCGA-61-1917-01 | 60 | Stage IIIB | G3 |
| TCGA-61-2009-01 | 65 | Stage IIIC | G3 |
| TCGA-31-1950-01 | 76 | Stage IIIC | G2 |
| TCGA-31-1951-01 | 58 | Stage IIIC | G3 |
| TCGA-61-1995-01 | 43 | Stage IIIC | G3 |
| TCGA-24-2297-01 | 56 | Stage IIIC | G3 |
| TCGA-24-1555-01 | 50 | Stage IIIC | G3 |
| TCGA-25-1322-01 | 62 | Stage IV | G3 |
| TCGA-24-1427-01 | 58 | Stage IIIC | G3 |
| TCGA-29-1698-01 | 53 | Stage IIIC | G3 |
| TCGA-23-1023-01 | 65 | Stage IIIC | G3 |
| TCGA-13-0924-01 | 45 | Stage IV | G3 |
| TCGA-24-1847-01 | 45 | Stage IV | G3 |
| TCGA-24-1843-01 | 66 | Stage IIIC | G3 |
| TCGA-25-1321-01 | 65 | Stage IIIC | G3 |
| TCGA-30-1891-01 | 61 | Stage IIIC | G2 |
| TCGA-25-1326-01 | 61 | Stage IIIC | G3 |
| TCGA-29-2414-01 | 75 | Stage IIIC | G2 |
| TCGA-09-1670-01 | 57 | Stage IIIA | G3 |
| TCGA-24-2033-01 | 87 | Stage IIIC | G3 |
| TCGA-29-2414-02 | 75 | Stage IIIC | G2 |
| TCGA-61-1741-01 | 76 | Stage IIIB | G3 |
| TCGA-31-1953-01 | 52 | Stage IIIC | G3 |
| TCGA-30-1855-01 | 61 | Stage IIIC | G3 |
| TCGA-25-1324-01 | 74 | Stage IIIC | G3 |
| TCGA-09-2056-01 | 62 | Stage IIIC | G3 |
| TCGA-29-1701-01 | 56 | Stage IIIC | G3 |
| TCGA-25-1633-01 | 64 | Stage IIIC | G3 |
| TCGA-25-2396-01 | 71 | Stage IIIC | G3 |
| TCGA-61-1914-01 | 65 | Stage IIIC | G3 |
| TCGA-61-2094-01 | 63 | Stage IIIC | G3 |
| TCGA-29-1705-01 | 47 | Stage IIIC | G2 |
| TCGA-36-1580-01 | 82 | Stage IIIC | G3 |
| TCGA-25-2401-01 | 64 | Stage IIIC | G3 |
| TCGA-5X-AA5U-01 | 61 | Stage IIC | GX |
| TCGA-13-0916-01 | 49 | Stage IIIC | G3 |
| TCGA-23-2084-01 | 45 | Stage IV | G3 |
| TCGA-36-1577-01 | 43 | Stage IIC | G2 |
| TCGA-24-1551-01 | 53 | Stage IIIC | G3 |
| TCGA-61-1998-01 | 48 | Stage IIIC | G3 |
| TCGA-20-1684-01 | 51 | Stage IIIC | G3 |
| TCGA-24-1556-01 | 50 | Stage IIB | G3 |
| TCGA-57-1582-01 | 50 | Stage IIIC | G3 |
| TCGA-04-1362-01 | 59 | Stage IIC | G3 |
| TCGA-61-2113-01 | 53 | Stage IIC | G3 |
| TCGA-24-1924-01 | 65 | Stage IIIC | G3 |
| TCGA-31-1946-01 | 30 | Stage IIIC | G3 |
| TCGA-29-1699-01 | 57 | Stage IIIC | G3 |
| TCGA-61-2002-01 | 46 | Stage IIIC | G3 |
| TCGA-24-2035-01 | 65 | Stage IIIC | G3 |
| TCGA-09-1674-01 | 79 | Stage IIIC | G3 |
| TCGA-29-1710-01 | 54 | Stage IIIC | G2 |
| TCGA-24-1436-01 | 57 | Stage IIIC | G3 |
| TCGA-13-1497-01 | 47 | Stage IIIC | G3 |
| TCGA-24-2271-01 | 55 | Stage IIIC | G3 |
| TCGA-24-1552-01 | 77 | Stage IIIC | G3 |
| TCGA-61-1724-01 | 47 | Stage IIIC | G3 |
| TCGA-20-1685-01 | 45 | Stage IIIC | G3 |
| TCGA-24-1423-01 | 61 | Stage IIIC | G3 |
| TCGA-25-2398-01 | 71 | Stage IIIC | G3 |
| TCGA-29-1763-01 | 43 | Stage IIC | G2 |
| TCGA-29-1703-01 | 56 | Stage IIIC | G2 |
| TCGA-10-0936-01 | 69 | Stage IIIC | G3 |
| TCGA-25-2409-01 | 71 | Stage IV | G3 |
| TCGA-30-1718-01 | 44 | Stage IIIC | G3 |
| TCGA-29-1711-01 | 45 | Stage IIIC | G2 |
| TCGA-61-2008-01 | 40 | Stage IIC | G2 |
| TCGA-61-1740-01 | 71 | Stage IIIC | G3 |
| TCGA-29-1785-01 | 55 | Stage IIIC | G3 |
| TCGA-13-1506-01 | 45 | Stage IIIC | G3 |
| TCGA-29-1781-01 | 69 | Stage IIIC | G3 |
| TCGA-36-1570-01 | 49 | Stage IIIC | G3 |
| TCGA-24-1930-01 | 53 | Stage IIIC | G3 |
| TCGA-13-0799-01 | 44 | Stage IIIC | G3 |
| TCGA-30-1862-01 | 65 | Stage IV | G2 |
| TCGA-23-1114-01 | 55 | Stage IIIC | G3 |
| TCGA-25-1626-01 | 65 | Stage IIIC | G3 |
| TCGA-61-2097-01 | 71 | Stage IIC | G2 |
| TCGA-24-1616-01 | 56 | Stage IIIC | G3 |
| TCGA-23-1109-01 | 62 | Stage IIIC | G3 |
| TCGA-29-2427-01 | 60 | Stage IIIC | G3 |
| TCGA-29-1695-01 | 62 | Stage IIIC | G2 |
| TCGA-61-1737-01 | 42 | Stage IV | G3 |
| TCGA-29-2425-01 | 60 | Stage IIIC | G2 |
| TCGA-09-1662-01 | 58 | Stage IV | G3 |
| TCGA-13-1512-01 | 49 | Stage IIIC | G3 |
| TCGA-29-1697-01 | 62 | Stage IIIC | G3 |
| TCGA-24-2024-01 | 72 | Stage IIIC | G3 |
| TCGA-57-1583-01 | 57 | Stage IIIC | G3 |
| TCGA-24-1562-01 | 67 | Stage IIIC | G3 |
| TCGA-57-1584-01 | 47 | Stage IIIC | G3 |
| TCGA-13-1411-01 | 81 | Stage IIIC | G3 |
| TCGA-61-2088-01 | 51 | Stage IIIC | G3 |
| TCGA-25-1625-01 | 66 | Stage IIIC | G3 |
| TCGA-24-2288-01 | 70 | Stage IIIC | G3 |
| TCGA-25-1623-01 | 71 | Stage IV | G3 |
| TCGA-25-1312-01 | 69 | Stage IV | G3 |
| TCGA-61-1725-01 | 40 | Stage IIIC | G3 |
| TCGA-25-2042-01 | 60 | Stage IIIC | G3 |
| TCGA-23-1026-01 | 45 | Stage IIIC | G3 |
| TCGA-29-1770-01 | 54 | Stage IIIC | G2 |
| TCGA-09-2054-01 | 58 | Stage IIIC | G3 |
| TCGA-61-1919-01 | 58 | Stage IIIC | G2 |
| TCGA-61-1900-01 | 51 | Stage IIIB | G3 |
| TCGA-24-1558-01 | 73 | Stage IIIC | G3 |
| TCGA-59-2354-01 | 63 | Stage IIIC | G3 |
| TCGA-09-2051-01 | 42 | Stage IIIC | G3 |
| TCGA-09-1667-01 | 61 | Stage IIC | G2 |
| TCGA-23-1809-01 | 63 | Stage IIC | G3 |
| TCGA-31-1956-01 | 60 | Stage IIIB | G3 |
| TCGA-13-0730-01 | 71 | Stage IIIC | G3 |
| TCGA-04-1519-01 | 48 | Stage IIIC | G3 |
| TCGA-25-1870-01 | 59 | Stage IIIC | G3 |
| TCGA-29-1688-01 | 39 | Stage IIIC | G2 |
| TCGA-61-2104-01 | 53 | Stage IIC | G2 |
| TCGA-13-0801-01 | 46 | Stage IIIC | G3 |
| TCGA-24-1928-01 | 77 | Stage IIIC | G3 |
| TCGA-36-1569-01 | 52 | Stage IIIC | G3 |
| TCGA-25-1315-01 | 50 | Stage IIIC | G3 |
| TCGA-24-1418-01 | 68 | Stage IIIC | G3 |
| TCGA-24-1428-01 | 50 | Stage IIIC | G3 |
| TCGA-23-2077-01 | 45 | Stage IIIC | G3 |
| TCGA-09-1669-01 | 54 | Stage IIIA | G3 |
| TCGA-24-1430-01 | 68 | Stage IIIC | G3 |
| TCGA-36-1581-01 | 63 | Stage IIC | G3 |
| TCGA-29-1776-01 | 63 | Stage IIIC | G3 |
| TCGA-29-1696-01 | 43 | Stage IIIC | G2 |
| TCGA-13-0913-01 | 53 | Stage IIIC | G3 |
| TCGA-09-0364-01 | 80 | Stage IIC | G3 |
| TCGA-61-2111-01 | 61 | Stage IV | G3 |
| TCGA-36-1571-01 | 53 | Stage IIIB | G3 |
| TCGA-24-2020-01 | 67 | Stage IIIC | G3 |
| TCGA-20-1687-01 | 46 | Stage IV | G3 |
| TCGA-24-2262-01 | 57 | Stage IIIC | G3 |
| TCGA-24-1850-01 | 72 | Stage IIIC | G3 |
| TCGA-25-1313-01 | 62 | Stage IV | G3 |
| TCGA-13-1489-02 | 70 | Stage IIIC | G2 |
| TCGA-29-1694-01 | 45 | Stage IIIC | GX |
| TCGA-23-1027-01 | 48 | Stage IIIC | G3 |
| TCGA-59-2355-01 | 58 | Stage IV | G3 |
| TCGA-24-1604-01 | 66 | Stage IIIC | G3 |
| TCGA-25-1319-01 | 73 | Stage IIIC | G3 |
| TCGA-24-1564-01 | 67 | Stage IIIC | G3 |
| TCGA-57-1994-01 | 63 | N/A | N/A |
| TCGA-29-1778-01 | 77 | Stage IIIC | G3 |
| TCGA-13-0899-01 | 60 | Stage IIIC | G3 |
| TCGA-24-2038-01 | 68 | Stage IIIA | GB |
| TCGA-13-0800-01 | 52 | Stage IIIC | G3 |
| TCGA-25-2399-01 | 80 | Stage IIIC | G3 |
| TCGA-24-1548-01 | 57 | Stage IIIC | G3 |
| TCGA-59-A5PD-01 | 55 | Stage IC | G3 |
| TCGA-25-2391-01 | 57 | Stage IIIC | G3 |
| TCGA-29-2428-01 | 58 | Stage IIIC | G3 |
| TCGA-25-1314-01 | 42 | Stage IV | G3 |
| TCGA-25-2393-01 | 81 | Stage IIIC | G3 |
| TCGA-23-2081-01 | 49 | Stage IV | G3 |
| TCGA-29-1762-01 | 59 | Stage IV | G2 |
| TCGA-23-1123-01 | 59 | Stage IIIC | G3 |
| TCGA-24-1435-01 | 57 | Stage IIIC | G3 |
| TCGA-24-2026-01 | 79 | Stage IIIC | G3 |
| TCGA-36-1575-01 | 83 | Stage IIIB | G3 |
| TCGA-10-0928-01 | 71 | Stage IIIC | G3 |
| TCGA-36-1568-01 | 52 | Stage IIIC | G3 |
| TCGA-36-1576-01 | 76 | Stage IIIC | G3 |
| TCGA-24-1463-01 | 70 | Stage IIIC | G3 |
| TCGA-57-1993-01 | 56 | Stage IIIC | G3 |
| TCGA-24-2036-01 | 50 | Stage IIIA | G3 |
| TCGA-13-1403-01 | 48 | Stage IIIC | G3 |
| TCGA-09-0369-01 | 56 | Stage IIIC | G3 |
| TCGA-24-1103-01 | 50 | Stage IIIC | G3 |
| TCGA-25-1871-01 | 70 | Stage IIIC | G3 |
| TCGA-23-1029-01 | 46 | Stage IIIC | G3 |
| TCGA-25-1630-01 | 73 | Stage IIIC | G3 |
| TCGA-25-1634-01 | 75 | Stage IIIC | G3 |
| TCGA-61-1721-01 | 38 | Stage IV | G1 |
| TCGA-24-1413-01 | 51 | Stage IIIC | G3 |
| TCGA-25-1323-01 | 72 | Stage IIIC | G3 |
| TCGA-61-2110-01 | 56 | Stage IIIC | N/A |
| TCGA-61-2109-01 | 40 | Stage IIIC | G3 |
| TCGA-04-1514-01 | 45 | Stage IIIA | G2 |
| TCGA-VG-A8LO-01 | 55 | Stage IV | GB |
| TCGA-24-1549-01 | 58 | Stage IIIB | G3 |
| TCGA-29-1784-01 | 55 | Stage IIIC | G3 |
| TCGA-13-A5FT-01 | 67 | Stage IIIC | G3 |
| TCGA-61-1736-01 | 45 | Stage IIIC | G3 |
| TCGA-OY-A56P-01 | 48 | Stage IIIB | G3 |
| TCGA-29-1690-01 | 66 | Stage IIIC | G2 |
| TCGA-29-1761-01 | 80 | Stage IIIC | G3 |
| TCGA-24-2281-01 | 68 | Stage IIA | G3 |
| TCGA-61-1738-01 | 60 | Stage IIIC | G3 |
| TCGA-25-2400-01 | 76 | Stage IIIC | G3 |
| TCGA-61-2098-01 | 62 | Stage IIIC | G2 |
| TCGA-59-2363-01 | 40 | Stage IIIA | G3 |
| TCGA-24-1567-01 | 54 | Stage IIIB | G3 |
| TCGA-61-1918-01 | 45 | Stage IV | G3 |
| TCGA-36-1574-01 | 48 | Stage IIIC | G3 |
| TCGA-24-2023-01 | 54 | Stage IIIA | G3 |
| TCGA-13-1511-01 | 52 | Stage IV | G3 |
| TCGA-25-1318-01 | 54 | Stage IIIC | G3 |
| TCGA-25-2392-01 | 75 | Stage IV | G3 |
| TCGA-24-1474-01 | 57 | Stage IIIC | G3 |
| TCGA-24-1846-01 | 45 | Stage IIIC | G3 |
| TCGA-09-1659-01 | 51 | Stage IIIC | G3 |
| TCGA-24-2027-01 | 51 | Stage IV | G3 |
| TCGA-29-1693-01 | 72 | Stage IIIC | G3 |
| TCGA-20-1682-01 | 56 | Stage IIIC | GX |
| TCGA-09-0366-01 | 55 | Stage IIIC | G3 |
| TCGA-24-1434-01 | 59 | Stage IIIC | G3 |
| TCGA-61-2003-01 | 53 | Stage IIIC | G2 |
| TCGA-24-1545-01 | 69 | Stage IIIC | G3 |
| TCGA-61-2012-01 | 81 | Stage IIC | G2 |
| TCGA-25-1632-01 | 68 | Stage IV | G3 |
| TCGA-24-2267-01 | 58 | Stage IIB | G3 |
| TCGA-24-2261-01 | 76 | Stage IIIC | G3 |
| TCGA-25-1316-01 | 55 | Stage IIIC | G3 |
| TCGA-09-1668-01 | 57 | Stage IIIC | G3 |
| TCGA-29-1770-02 | 54 | Stage IIIC | G2 |
| TCGA-61-1733-01 | 71 | Stage IIIC | G3 |
| TCGA-3P-A9WA-01 | 55 | Stage IIB | G3 |
| TCGA-24-1425-01 | 45 | Stage IIIC | G3 |
| TCGA-29-1691-01 | 51 | Stage IIIC | G2 |
| TCGA-61-2101-01 | 55 | Stage IIIC | G2 |
| TCGA-59-2352-01 | 78 | Stage IIIC | G3 |
| TCGA-OY-A56Q-01 | 78 | Stage IIA | G3 |
| TCGA-04-1365-01 | 87 | Stage IIIB | G3 |
| TCGA-24-1563-01 | 66 | Stage IIIC | G3 |
| TCGA-13-0897-01 | 54 | Stage IIIC | G3 |
| TCGA-24-2019-01 | 46 | Stage IIIC | G3 |
| TCGA-36-1578-01 | 63 | Stage IV | G3 |
| TCGA-61-2016-01 | 51 | Stage IIIC | G3 |
| TCGA-13-1498-01 | 73 | Stage IIIC | G3 |
| TCGA-24-1842-01 | 49 | Stage IIIC | G3 |
| TCGA-13-1405-01 | 49 | Stage IV | G3 |
| TCGA-24-2298-01 | 55 | Stage IIIC | G3 |
| TCGA-25-1328-01 | 38 | Stage IIIC | G3 |
| TCGA-30-1860-01 | 58 | Stage IIIC | G3 |
| TCGA-25-1631-01 | 73 | Stage IIIC | G3 |
| TCGA-13-0893-01 | 48 | Stage IIIC | G3 |
| TCGA-23-1122-01 | 53 | Stage IIIC | G3 |
| TCGA-13-2060-01 | 51 | Stage IV | G3 |
| TCGA-13-0890-01 | 56 | Stage IIIC | G3 |
| TCGA-59-2350-01 | 44 | Stage IV | G3 |
| TCGA-WR-A838-01 | 72 | Stage IIIC | G3 |
| TCGA-24-1467-01 | 51 | Stage IIIC | G3 |
| TCGA-09-1673-01 | 50 | Stage IV | G3 |
| TCGA-24-1544-01 | 71 | Stage IIIC | G3 |
| TCGA-04-1364-01 | 61 | Stage IIIC | G3 |
| TCGA-61-2102-01 | 74 | Stage IIIC | G3 |
| TCGA-24-1550-01 | 49 | Stage IIIC | G3 |
| TCGA-24-1419-01 | 62 | Stage IIIC | G3 |
| TCGA-24-1849-01 | 80 | Stage IIIC | G3 |
| TCGA-25-1320-01 | 65 | Stage IIIC | G3 |
| TCGA-30-1861-01 | 74 | Stage IIIC | G3 |
| TCGA-13-1410-01 | 57 | Stage IV | GX |
| TCGA-25-1627-01 | 73 | Stage IIIC | G3 |
| TCGA-30-1857-01 | 64 | Stage IV | G3 |
| TCGA-13-0920-01 | 65 | Stage IIIC | G3 |
| TCGA-09-0367-01 | 67 | Stage IIIC | G3 |
| TCGA-24-1416-01 | 34 | Stage IV | G3 |
| TCGA-24-0975-01 | 58 | Stage IIIC | G3 |
| TCGA-24-2293-01 | 47 | N/A | G3 |
| TCGA-61-1743-01 | 53 | Stage IIC | G2 |
| TCGA-31-1944-01 | 47 | Stage IIIC | G3 |
| TCGA-25-1317-01 | 66 | Stage IIIC | G3 |
| TCGA-25-1635-01 | 71 | Stage IIIC | G3 |
| TCGA-29-A5NZ-01 | 66 | Stage IIIC | G3 |
| TCGA-25-1628-01 | 67 | Stage IIIC | G3 |
| TCGA-24-1553-01 | 53 | Stage IIIB | G3 |
| TCGA-61-2092-01 | 57 | Stage IIIC | G3 |
| TCGA-25-1329-01 | 76 | Stage IIIC | G3 |
| TCGA-24-1844-01 | 64 | Stage IIIC | G3 |
| TCGA-24-1603-01 | 53 | Stage IIIB | G3 |
| TCGA-09-2044-01 | 77 | Stage IIB | G3 |
| TCGA-61-2008-02 | 40 | Stage IIC | G2 |
| TCGA-23-2078-01 | 66 | Stage IIIC | G3 |
| TCGA-20-1683-01 | 65 | Stage IIIC | G3 |
| TCGA-13-1505-01 | 63 | Stage IIIC | G3 |
| TCGA-24-2254-01 | 66 | Stage IIIC | G3 |
| TCGA-23-1120-01 | 60 | Stage IIIC | G3 |
| TCGA-24-1546-01 | 46 | Stage IIIC | G3 |
| TCGA-24-1557-01 | 49 | Stage IIIC | G3 |
| TCGA-59-2351-01 | 51 | Stage IIIC | G3 |
| TCGA-24-2289-01 | 68 | Stage IV | G3 |
| TCGA-13-1481-01 | 76 | Stage IIIC | G2 |
| TCGA-25-2397-01 | 59 | Stage IV | G3 |
| TCGA-09-2045-01 | 50 | Stage IV | G2 |
| TCGA-24-2290-01 | 56 | Stage IIIC | G3 |
| TCGA-61-2000-01 | 67 | Stage IIIC | G3 |
| TCGA-09-2048-01 | 63 | Stage IIIC | G3 |
| TCGA-61-1907-01 | 63 | Stage IIIC | G3 |
| TCGA-23-1111-01 | 63 | Stage IIIC | G3 |
| TCGA-31-1959-01 | 49 | Stage IV | G2 |
| TCGA-24-1560-01 | 51 | Stage IIIC | G4 |
| TCGA-29-1783-01 | 58 | Stage IIIC | G3 |
| TCGA-59-2348-01 | 59 | Stage IIIC | G3 |
| TCGA-24-1424-01 | 67 | Stage IIIC | G3 |
| TCGA-13-1507-01 | 77 | Stage IIIC | G3 |
| TCGA-25-2404-01 | 38 | Stage IIIC | G3 |
| TCGA-61-2095-01 | 54 | Stage IIIC | G2 |
| TCGA-29-1702-01 | 84 | Stage IIIC | G3 |
| TCGA-24-1417-01 | 54 | Stage IV | G3 |
| TCGA-57-1585-01 | 57 | Stage IIIC | G3 |
